# Knee joint distraction compared with high tibial osteotomy: a randomized controlled trial

**DOI:** 10.1007/s00167-016-4131-0

**Published:** 2016-04-22

**Authors:** J. A. D. van der Woude, K. Wiegant, R. J. van Heerwaarden, S. Spruijt, P. M. van Roermund, R. J. H. Custers, S. C. Mastbergen, F. P. J. G. Lafeber

**Affiliations:** 1 Limb and Knee Reconstruction Unit, Department of Orthopedic Surgery, Maartenskliniek Woerden, Woerden, The Netherlands; 2 Rheumatology and Clinical Immunology, University Medical Center Utrecht, F02.217, PO Box 85500, 3508 GA Utrecht, The Netherlands; 3 Department of Orthopedics, UMC Utrecht, Utrecht, The Netherlands

**Keywords:** Joint distraction, Knee osteoarthritis, High tibial osteotomy, Cartilage repair

## Abstract

**Purpose:**

Both, knee joint distraction as a relatively new approach and valgus-producing opening-wedge high tibial osteotomy (HTO), are knee-preserving treatments for knee osteoarthritis (OA). The efficacy of knee joint distraction compared to HTO has not been reported.

**Methods:**

Sixty-nine patients with medial knee joint OA with a varus axis deviation of <10° were randomized to either knee joint distraction (*n* = 23) or HTO (*n* = 46). Questionnaires were assessed at baseline and 3, 6, and 12 months. Joint space width (JSW) as a surrogate measure for cartilage thickness was determined on standardized semi-flexed radiographs at baseline and 1-year follow-up.

**Results:**

All patient-reported outcome measures (PROMS) improved significantly over 1 year (at 1 year *p* < 0.02) in both groups. At 1 year, the HTO group showed slightly greater improvement in 4 of the 16 PROMS (*p* < 0.05). The minimum medial compartment JSW increased 0.8 ± 1.0 mm in the knee joint distraction group (*p* = 0.001) and 0.4 ± 0.5 mm in the HTO group (*p* < 0.001), with minimum JSW improvement in favour of knee joint distraction (*p* = 0.05). The lateral compartment showed a small increase in the knee joint distraction group and a small decrease in the HTO group, leading to a significant increase in mean JSW for knee joint distraction only (*p* < 0.02).

**Conclusion:**

Cartilaginous repair activity, as indicated by JSW, and clinical outcome improvement occurred with both, knee joint distraction and HTO. These findings suggest that knee joint distraction may be an alternative therapy for medial compartmental OA with a limited mechanical leg malalignment.

**Level of evidence:**

Randomized controlled trial, Level I.

## Introduction

Historically, the treatment of knee osteoarthritis (OA) was limited to total knee arthroplasty (TKA) in the event of conservative treatment failure. However, there is an increasing recognition that even in advanced knee OA, joint repair may occur. For example, high tibial osteotomy (HTO) is a well-established surgical procedure for medial compartment knee OA in varus malalignment [26, 27] with an 87–99 % 5-year survival and a 66–84 % 10-year survival [6, 7, 10, 13, 34] and can thus defer TKA in OA. Evidence for intrinsic cartilage repair after opening-wedge HTO is sparse. Four studies evaluated cartilage quality after opening-wedge HTO by second-look arthroscopic assessment. Jung et al. performed two retrospective, sequential reviews. In the first, they found partial coverage of the medial femoral condyle in 92 % of the knees, but only maturation in 4 % of the knees 2 years after HTO [17]. In the second study, two groups were compared: one group was treated with HTO alone, and in the other group, HTO was combined with subchondral bone drilling. Grade II fibrocartilage formation in both groups was equal (90 % in HTO vs. 94 % with additional drilling) [18]. Spahn et al. [42] reported, one and a half years after HTO, restoration of deep cartilage lesions in 60 %. Koh et al. [20] compared HTO with additional mesenchymal stem cell therapy or plasma therapy. Evaluation showed partial or even fibrocartilage coverage in 50 % of the patients with additional mesenchymal stem cell therapy, but in only 10 % of the patients in the plasma group.

Knee joint distraction is a more recently developed surgical joint-preserving treatment that also appears to be associated with joint tissue repair. Joint distraction for OA has been reported for several joints including the knee [1, 2, 11, 12, 15, 31]. Only one of these studies prospectively evaluated patients [15]; however, all studies showed radiographic joint space width (JSW) improvement. The first prospective open uncontrolled study reported substantial clinical improvement and cartilage repair by knee joint distraction resulting in the planned TKA being postponed for at least 5 years [15, 23, 45]. This was associated with MRI-determined cartilaginous repair 2 years later and associated increased radiographic JSW under weight-bearing conditions [45]. The increase in JSW was maintained at 5 years as compared to the natural progression of cartilage loss [23].

Both knee joint distraction and HTO are based on unloading of the affected joint compartment cartilage, which is thought to be beneficial in OA [24]. The therapeutic rationale is that abnormal loading is a major cause of OA development and progression, and joint unloading may slow or prevent OA progression, or even lead to repair. Because both HTO and knee joint distraction make use of (partial/temporarily) joint unloading, both are associated with JSW improvement, and both reported to result in prolonged clinical benefit, we compared these treatments in a randomized controlled trial. It was hypothesized that there was no clinical important difference in efficacy between knee joint distraction and HTO treatment.

## Materials and methods

The 69 patients with medial knee compartmental OA were recruited between 2011 and 2013 in this prospective, two-centre, randomized controlled trial comparing HTO with knee joint distraction. Fifty-five patients were included at the Maartenskliniek Woerden, and fourteen patients were included at the University Medical Center Utrecht. Randomization of 2:1 for HTO versus knee joint distraction was performed in blocks of six at each of the institutes using standard randomization software. In order to minimize the number of knee joint distraction treatments, the medical ethics committee, considering knee joint distraction an experimental treatment, obligated this randomization ratio. This resulted in 46 patients randomized to HTO, and 23 to knee joint distraction.

Patients and physicians were aware of treatment assignment after allocation. Inclusion criteria were OA of the medial compartment of the knee with a tibiofemoral angle of less than 10° of varus, age <65 years, intact knee ligaments, normal range of motion (minimum of 120° flexion) and a body mass index (BMI) <35. Patients with contralateral knee OA needing treatment were excluded, as were those with primary patellofemoral OA, bi-compartmental OA, a history of inflammatory or septic arthritis, a (partial) lateral meniscectomy, inability to cope with an external fixator, complete joint space absence on X-ray, post-traumatic fibrosis due to a fracture of the tibial plateau, inability to undergo MRI examination or previous surgery on the same knee within the past 6 months.

### Treatments

In HTO, the goal was to shift the weight-bearing line laterally, with the post-operative mechanical axis running laterally through the tibial plateau, at 62 % of its entire width (measured from the medial side). Using standing whole leg radiographs, the amount of needed correction was determined using the Miniaci method [33]. At the Maartenskliniek Woerden, a specialized osteotomy clinic, two experienced surgeons (RH, SS) performed 36 HTO’s. At the University Medical Center Utrecht, one experienced surgeon (PR) performed nine HTO’s. Bi-plane medial-based opening-wedge osteotomy was performed, including a distal release of the superficial fibres of the medial collateral ligament. TomoFix medial high tibial plates and screws (DePuy Synthes, Switzerland) or Synthes locking compression plate (LCP) system (DePuy Synthes, Switzerland) were used for fixation. In three cases, in the University Medical Center Utrecht, autologous iliac bone grafts were applied to fill the osteotomy gap. Post-operative partial weight bearing (maximum of 20 kg) was allowed for 6 weeks; thereafter, all patients started gradual full weight bearing. Subcutaneous low molecular weight heparin thromboembolism prophylaxis was used for 6 weeks.

**Fig. 1:**
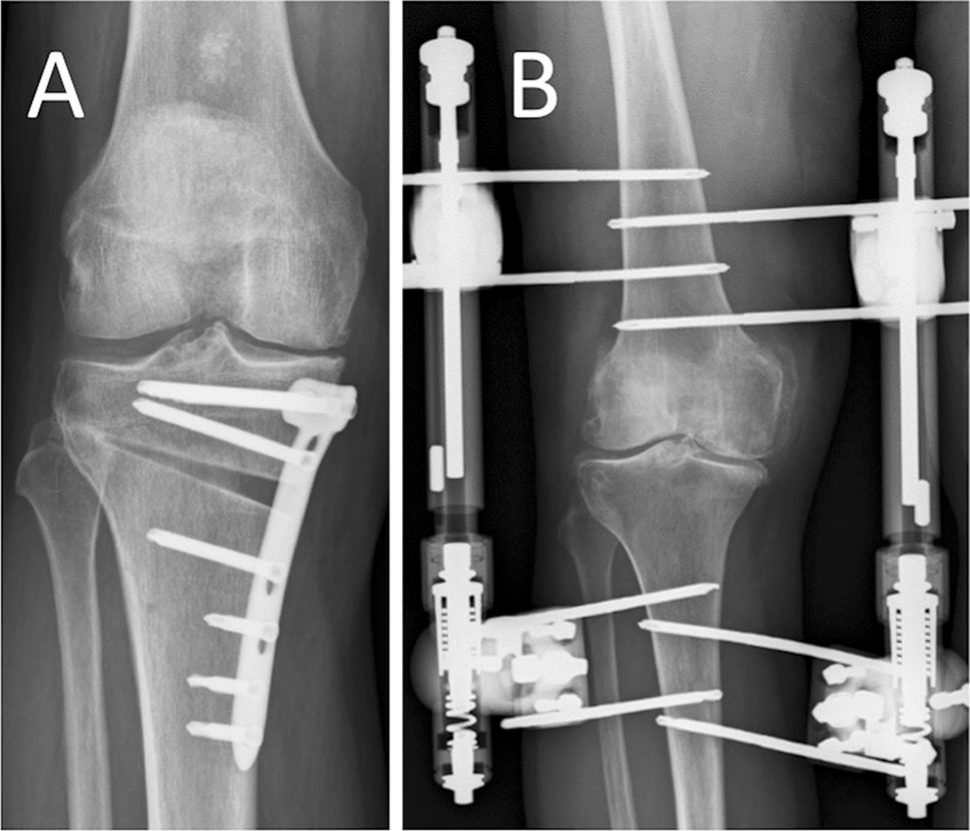
Example of a post-operative radiograph, **a** patient treated with HTO, **b** patient treated with knee joint distraction

Knee joint distraction was performed by use of a proof of concept external distraction device, normally used for bone lengthening or fracture stabilization. Two dynamic monotubes (Triax, Stryker, 45 kg spring with 2.5 mm displacement) were fixed in a standard fashion to bone pins, two for each of the four locations (lateral and medial for femur and tibia; see Fig. 1), bridging the knee joint at the lateral and medial side. Intra-operatively, the tubes were distracted 2 mm. Post-operatively, every day the tubes were 1 mm distracted, until 5-mm distraction was reached. At day 4, distraction was checked by weight-bearing radiographs and adapted if needed. Hereafter, patients were discharged from the hospital and allowed full weight bearing with crutches (for stability). At 3 weeks, patients visited the outpatient department for radiographic evaluation of the distraction and pin tract evaluation. After 6 weeks (average duration 43 days, range 39–50 days), the frame and pins were surgically removed. Partial weight bearing (maximum 20 kg) was allowed, and patients were discharged the same day. Gradually, they regained normal full loading in approximately 6 weeks (expansion of 15 kg every week). Low molecular weight heparin as thrombosis prophylaxis was given for 9 weeks (during distraction treatment and for 3 weeks after frame removal).

### Clinical outcome

The Western Ontario and McMaster Universities Osteoarthritis Index (WOMAC, version 3.1) and the validated Dutch knee injury and osteoarthritis outcome score (KOOS) were used to score clinical improvement, normalized to a 100-point scale; 100 being the best condition. Both questionnaires were used to make comparison with other studies, using either of the two, possible. The intermittent and constant osteoarthritis pain score (ICOAP) for the knee was the secondary clinical outcome parameter (0–100, 0 meaning no pain). A visual analogue scale for pain (VAS pain; 0–100 mm, 0 meaning no pain) was the tertiary clinical outcome parameter. The EQ-5D-3L was used to assess improvement of quality of life. The obtained questionnaire was transformed to an EQ-5D index score (0–1, 1 being the best). The Short Form 36 (SF-36) health survey was used to measure the health status of the patients. The SF-36 items were transformed to the physical (PCS) and mental (MCS) component summary score. At baseline and 3, 6, and 12 months, the KOOS/WOMAC questionnaire, ICOAP questionnaire, the VAS pain, and the EQ-5D-3L questionnaire were assessed. At baseline and 6 and, 12 months, the SF-36 was assessed.

### Structural outcome

To assess structural outcome, knee radiographs were obtained at baseline and 12 months post-operatively. The knee images were standardized weight-bearing, semi-flexed posterior–anterior radiographic views according to the protocol of Buckland-Wright and were evaluated by the use of knee images digital analyses (KIDA) validated software [30]. This is a fully mathematical method to analyse the mean and minimum joint space width (JSW) of the knee. The minimum JSW was measured as the shortest distance between the femur and the tibia. The mean JSW of the medial compartment is defined as the mean of four predefined locations. In case of possible magnification of the radiograph, an aluminium step-wedge is used for correction. The method has frequently been used and reported on, inter-observer reproducibility is very high (*R* = 0.85–0.90), and the intra-observer variation (ICC = 0.73–0.99) good [19, 30]. Image analyses were performed blinded to the order of acquisition and patient characteristics. The mean and minimum JSWs are given for the medial and lateral compartment in millimetre, rounded to one decimal. No MRI analyses were performed at 1 year because the presence of the plates (removed after 18 months) in the HTO group.

The medical ethics committee of the University Medical Center Utrecht approved this level I, prospectively, randomized, controlled study (No. 11/072), the site-specific institutional review boards of the Maartenskliniek Woerden and University Medical Center Utrecht approved the study protocol before study initiation, and it was registered on the Netherlands National Trial Register (NTR2900). All patients provided written informed consent before enrolment.

### Statistical analyses

A sample size calculation was performed based on non-inferiority using a power of 80 % [47]. To account for possible dropout and/or insufficient data quality, the sample size was increased by 15 %. Two-sided paired tests (normally distributed data sets) were used to evaluate whether the follow-up values differed from the baseline values. To compare the changes between 1 year and baseline between both groups, independent samples *t* test was used (normally distributed data sets). For difference between Kellgren and Lawrence grade, Chi-square test for trend was used. Tests were two-sided, and probability *p* < 0.05 was considered statistically significant. SPSS software version 22.0 was used to perform statistical analyses.

## Results

**Fig. 2:**
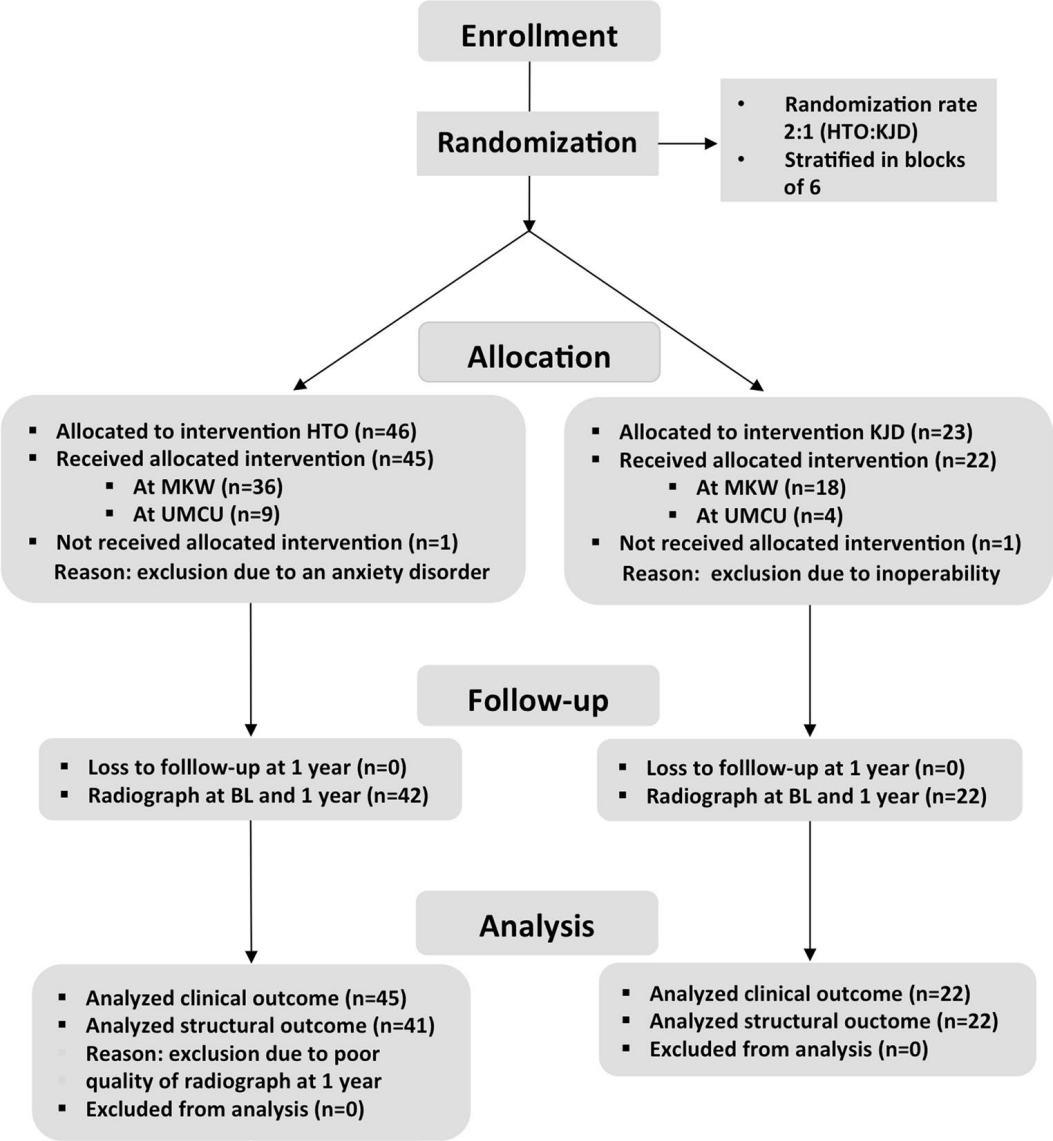
Flow chart including the numbers of excluded patients, as well allocation of the randomized treatment and the analysed patients per treatment arm. *KJD* knee joint distraction, *MKW* Maartenskliniek Woerden, *UMCU* University Medical Center Utrecht

Of the 69 randomized patients enrolled, 23 were assigned to knee joint distraction and 46 to HTO. After randomization, one knee joint distraction and one HTO assigned patient were excluded (see Fig. 2). Of the remaining 67 patients, the baseline characteristics and an overview of previous knee surgery of the affected knee are given in Table 1. In the HTO group, the mean mechanical tibiofemoral axis was preoperatively 6.2° ± 2.3° (mean ± SD) of varus and post-operatively 2.4° ± 1.8° of valgus. The mean medial proximal tibia angle changed from 86.5° ± 1.9° preoperatively to 94.0° ± 4.7° post-operatively.

**Table 1:** Baseline characteristics

Characteristics	High tibial osteotomy	Knee joint distraction	
Mean (±SEM)	(*n* = 45)	(*n* = 22)	*p* value
Male gender (*n*)	27/45 (60 %)	16/22 (73 %)	n.s.
Height (cm)	177 ± 2	178 ± 2	n.s.
Weight (kg)	85.2 ± 2.1	87.2 ± 2.8	n.s.
Body mass index (kg/m^2^)	27.2 ± 0.5	27.5 ± 0.7	n.s.
Affected knee (left)	20/45 (44 %)	10/22 (45 %)	n.s.
Age at surgery (year)	49.4 ± 1.0	51.2 ± 1.1	n.s.
Kellgren and Lawrence (median)	3	3	n.s.
Grade 0 (*n*)	1 (2 %)	0 (0 %)	
Grade 1 (*n*)	5 (11 %)	6 (27 %)	
Grade 2 (*n*)	12 (27 %)	4 (18 %)	
Grade 3 (*n*)	23 (51 %)	11 (50 %)	
Grade 4 (*n*)	4 (9 %)	1 (5 %)	
Tibiofemoral axis (°)	6.2 ± 0.3	5.8 ± 0.6	n.s.
**Previous surgery**			
**Operation **(**number**)			
ACL reconstruction (*n*)	4	2	
High tibial osteotomy (*n*)	2	0	
Arthroscopy	31	16	
Partial meniscectomy (*n*)	18	12	
Arthroscopic joint lavage (*n*)	13	4	
Open medial meniscectomy (*n*)	3	1	
Tibial crest transposition (*n*)	1	0	
Fixation osteochondritis dissecans lesion (*n*)	1	0	

### Clinical outcome

**Fig. 3:**
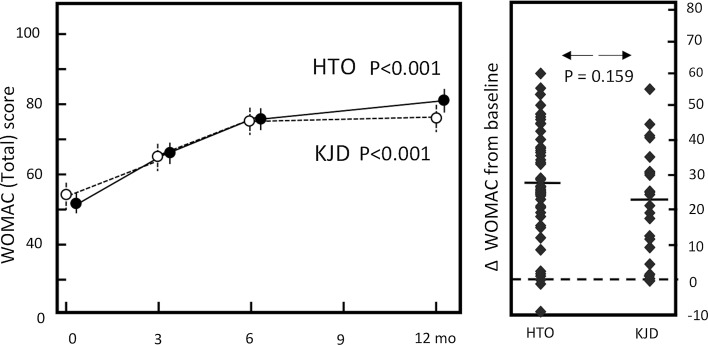
WOMAC total. *Dotted line* represents the knee joint distraction group (*n* = 22), *solid line* represents the HTO group (*n* = 45). Mean values ± SEM are given. *p* values show statistical difference in values at 1-year follow-up compared to pre-treatment values. Mean change of WOMAC total (*right*): for both groups (average:* dash*) and for every individual patient (*squares*)

**Fig. 4:**
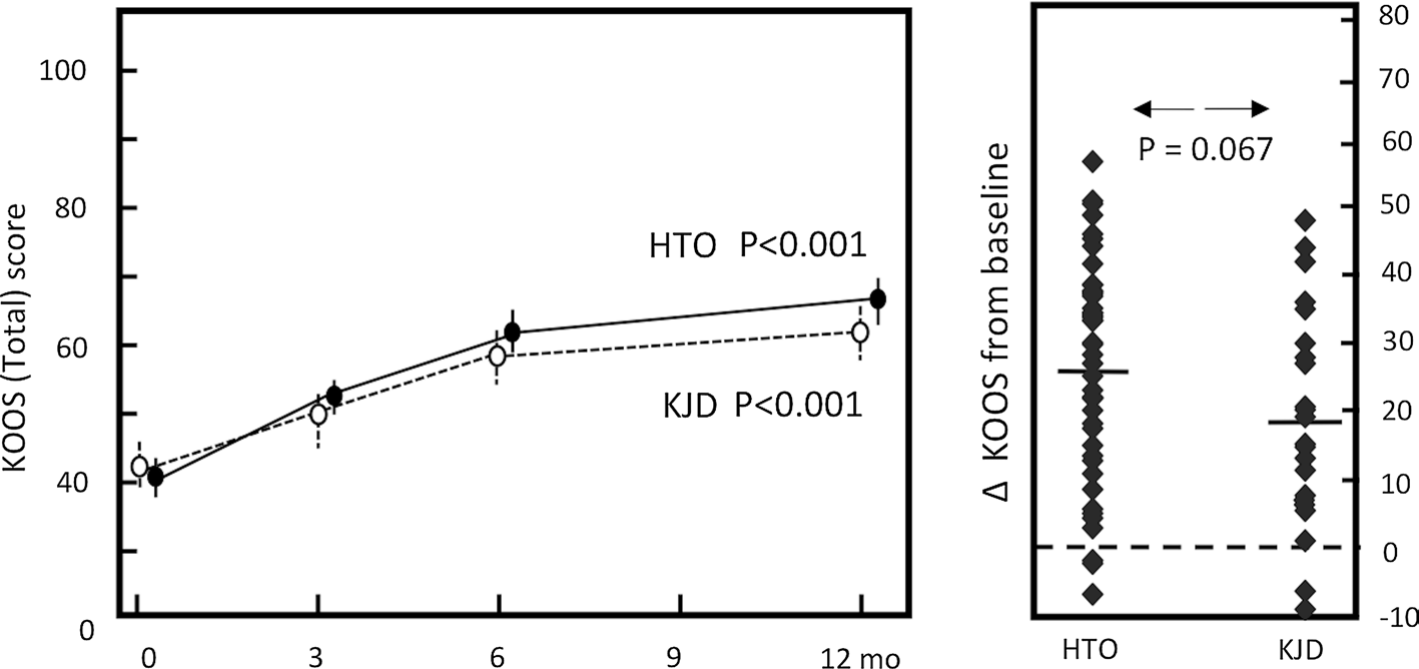
KOOS total. *Dotted line* represents the knee joint distraction group (*n* = 22), *solid line* represents the HTO group (*n* = 45). Mean values ± SEM are shown. *p* values show statistical difference in values at 1-year follow-up compared to pre-treatment values. Mean change of KOOS total score (*right*): for both groups (average:* dash*) and for every individual patient (*squares*)

A clear clinical improvement, based on the total WOMAC score (Fig. 3) and KOOS (Fig. 4), was noted in both groups. For the five subscales of the KOOS, the three individual components of the WOMAC index, the ICOAP for the knee, the physical component scale (PCS) of the SF-36, the VAS pain score and the EQ-5D similar improvements were found (Table 2).

**Table 2:** KOOS, WOMAC and SF-36 scores pre-operative and at the post-operative follow-up moments for both groups

		High tibial osteotomy				Knee joint distraction					
Score (±SD)		BL	3 m	6 m	12 m	BL → 1Y	BL	3 m	6 m	12 m	BL → 1Y
KOOS (0–100)	Pain	46 ± 18	64 ± 17*	72 ± 19*	77 ± 19*	32 ± 19	47 ± 18	61 ± 22*	71 ± 21*	72 ± 18*	25 ± 19
	Symptom	54 ± 15	63 ± 14*	71 ± 15*	73 ± 16*	19 ± 18	56 ± 19	54 ± 20	63 ± 20	68 ± 19*	13 ± 17
	ADL	54 ± 17	68 ± 14*	76 ± 18*	82 ± 17*	28 ± 17	55 ± 21	65 ± 22	75 ± 19*	78 ± 19*	24 ± 17
	Sport/rec	23 ± 19	31 ± 22*	47 ± 25*	53 ± 31*	29 ± 24	29 ± 19	36 ± 30	44 ± 25*	49 ± 27*	21 ± 24
	QOL	27 ± 15	37 ± 17*	47 ± 21*	55 ± 25*	28 ± 19^#^	32 ± 16	30 ± 17	43 ± 21*	44 ± 20*	12 ± 17
	Total	41 ± 13	53 ± 14*	63 ± 17*	68 ± 19*	27 ± 16	43 ± 17	50 ± 18	60 ± 18*	62 ± 18*	19 ± 16
WOMAC (0–100)	Pain	50 ± 21	68 ± 17*	76 ± 19*	81 ± 18*	31 ± 19	54 ± 21	69 ± 23*	76 ± 20*	76 ± 15*	23 ± 21
	Stiffness	46 ± 21	58 ± 21*	64 ± 23*	69 ± 19*	22 ± 21^#^	50 ± 20	56 ± 21	58 ± 19	60 ± 18	10 ± 24
	Function	54 ± 17	68 ± 14*	76 ± 18*	82 ± 17*	28 ± 17	55 ± 21	65 ± 22	75 ± 19*	78 ± 19*	24 ± 17
	Total	52 ± 17	67 ± 14*	75 ± 17*	81 ± 16*	29 ± 17	54 ± 20	65 ± 20	74 ± 18*	76 ± 17*	22 ± 16
ICOAP (100–0)	Constant	48 ± 21	34 ± 24*	29 ± 27*	19 ± 24*	−29 ± 24	50 ± 20	35 ± 31	24 ± 25*	23 ± 20*	−27 ± 21
	Intermittent	54 ± 22	33 ± 22*	35 ± 26*	23 ± 24*	−31 ± 31	50 ± 20	38 ± 29	34 ± 24*	34 ± 22*	−17 ± 20
	Combined	51 ± 20	34 ± 21*	32 ± 26*	21 ± 23*	−30 ± 26	50 ± 20	36 ± 29	30 ± 24*	29 ± 19*	−22 ± 18
VAS (0–100)	Pain	65 ± 21	47 ± 26*	37 ± 24*	27 ± 23*	−38 ± 26^#^	55 ± 24	46 ± 29	34 ± 23*	36 ± 26*	−19 ± 26
EQ-5D (0–1)	Index score	0.64 ± .2	0.68 ± .2^#^	0.68 ± .3	0.79 ± .3*	0.15 ± .3	0.63 ± .2	0.52 ± .3	0.69 ± .2	0.77 ± .1*	0.14 ± .3
SF-36	PCS	36 ± 8		42 ± 10*	46 ± 10*	10 ± 9^#^	37 ± 7		40 ± 10	42 ± 10*	5 ± 8
	MCS	55 ± 8		53 ± 11	54 ± 10	−1 ± 8	56 ± 8		56 ± 8	54 ± 9	−1 ± 9
Flexion (°)	Knee	132 ± 8	127 ± 10	128 ± 8	128 ± 8		130 ± 7	115 ± 17	128 ± 9	132 ± 8	

* *p* < 0.05 relative to the preoperative score

^#^ *p* < 0.05 difference between HTO and knee joint distraction

The HTO group showed statistically significantly greater improvements in the mean change of the KOOS subscale quality of life (*p* = 0.002), the WOMAC subscale stiffness (*p* = 0.028), the VAS pain score (*p* = 0.006) and SF-36 PCS (*p* = 0.024).

Knee flexion in both the knee joint distraction and the HTO group equalled to baseline levels (132° in the knee joint distraction group and 128° in the HTO group) at 12-month follow-up. After an initial fall in joint flexion, levels returned to baseline levels after 6 months of knee joint distraction.

### Structural outcome

**Fig. 5:**
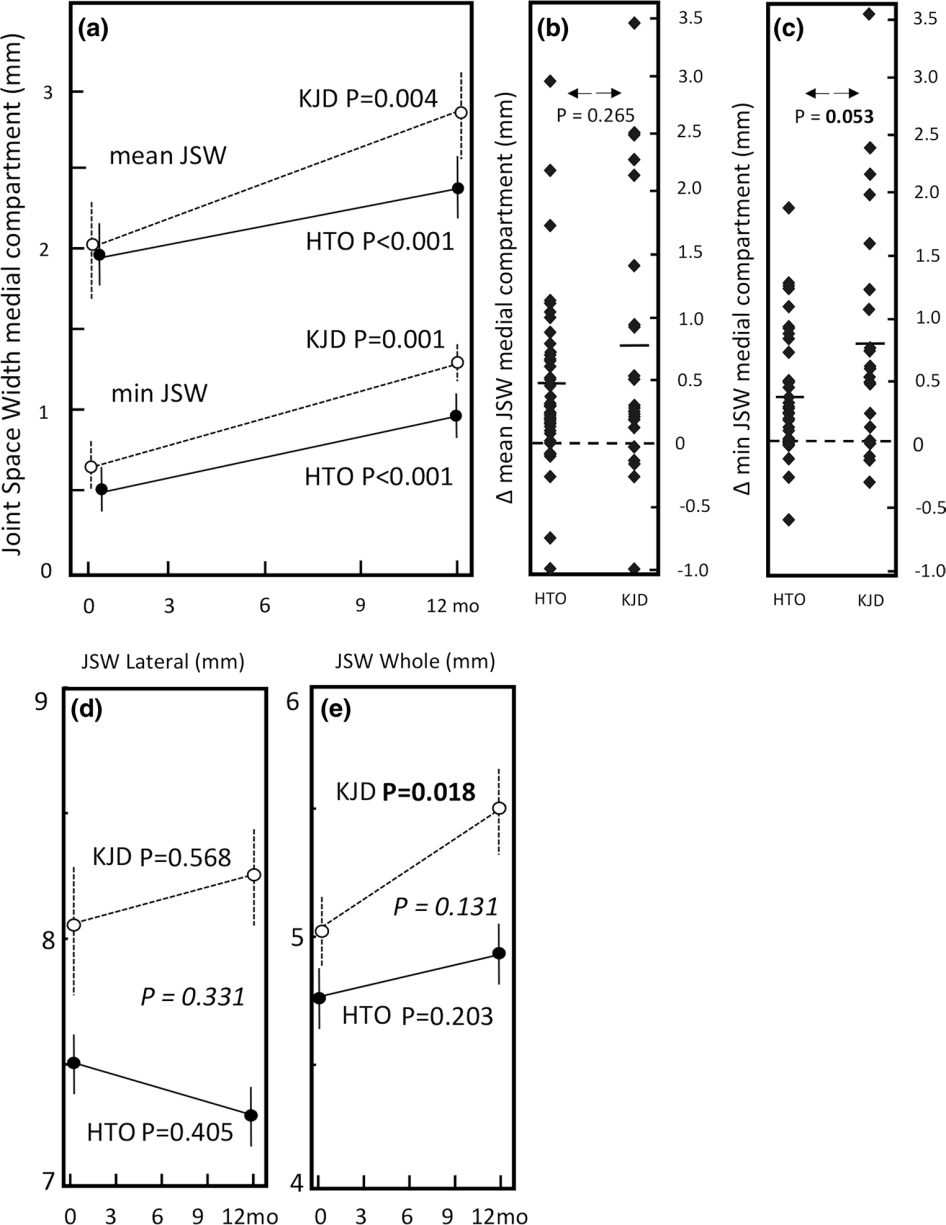
**a** Mean quantitative radiograph analyses of the medial (affected) compartment of both treatment groups. The *solid line* represents HTO group (*n* = 41), and the *dotted line* represents the knee joint distraction group (*n* = 22). Mean values ± SEM are presented. *p* values show statistical difference in values at 1-year follow-up compared to pre-treatment values. **b** Mean change of mean JSW of medial compartment. For both groups (average:* dash*) and for every individual patient (*squares*). **c** Mean change of minimum JSW of medial compartment. For both groups (average:* dash*) and for every individual patient (*squares*). **d** Mean JSW of the lateral (least affected) compartment of both treatment groups. **e** Mean JSW of the whole joint of the both treatment groups. The *p* value in italic shows statistical difference in change over 1 year between the two treatment groups

The mean JSW of the medial compartment in the knee joint distraction group increased significantly from 2.0 ± 1.4 mm (mean ± SD) towards 2.8 ± 1.3 mm at 1 year (*p* = 0.004), whereas the minimum JSW increased 0.8 ± 1.0 mm (*p* = 0.001). In the HTO group, both the mean and minimum medial compartment showed a less striking trend with the mean JSW increasing from 2.0 ± 1.2 mm at baseline to 2.4 ± 1.3 mm at 1 year (*p* < 0.001). The minimum JSW increased on average 0.4 ± 0.5 mm (*p* < 0.001). See also Fig. 5a–c.

The mean HTO lateral compartment JSW (Fig. 5d) showed a decline of 0.2 ± 1.3 mm (n.s.), whereas the knee joint distraction group showed an increase of 0.2 ± 1.4 mm (n.s.). The mean JSW of the whole joint (medial and lateral compartment averaged) increased 0.5 ± 0.9 mm in the knee joint distraction group (*p* = 0.018) and showed no significant increase in the HTO group (+0.2 ± 0.8 mm, see also Fig. 5e).

The increase in the minimum JSW neared statistical significant difference between the two groups (*p* = 0.053), whereas the increase in the mean JSW of the medial compartment and of the whole joint showed no statistical significant difference between the two groups.

### Adverse events

An overview of the adverse events is given in Table 3. In the knee joint distraction group, thirteen patients (59 %) had single or multiple pin tract infections, nine of which were treated adequately with oral antibiotics. In the HTO group, two patients (4.4 %) had a post-operative wound infection.

**Table 3:** Overview of adverse events

**Knee joint distraction**	
Pin tract infection	
*Antibiotics oral*	9
*Antibiotics intravenous*	3
*with surgical irrigation and debridement*	2
Osteomyelitis (3 weeks post-frame removal)	
* Antibiotics intravenous with surgical irrigation and debridement*	1
Monotube failure (re-fixation)	1
Breaking of bone pin during fixation	1
Manipulation knee under anaesthesia (17 days after removal frame)	1
**High tibial osteotomy**	
Wound infection	
*Antibiotics oral*	1
*Antibiotics intravenous*	1
Erysipelas	
*Antibiotics intravenous*	1
Partial medial meniscectomy (affected knee, <6 months)	1

## Discussion

This study showed that knee joint distraction was non-inferior to HTO for clinical symptoms and for JSW improvement in knee joint OA. In addition, in both treatment groups, all the domains of the KOOS, the EQ-5D index and the SF-36 PCS subscale demonstrated significant improvements at 1-year follow-up. Cartilage repair activity as indicated by JSW on radiographs was slightly better for knee joint distraction, whereas clinical parameters improved slightly more in the case of HTO.

This is the first study comparing knee joint distraction in randomized set-up with another knee joint-preserving surgical strategy. With respect to knee joint distraction, one previous prospective uncontrolled study of twenty patients showed efficacy [15, 45]. In the present study, patients treated with knee joint distraction showed similar outcome at 1-year follow-up on the WOMAC (76 ± 17, *n* = 22 and 77 ± 21, *n* = 20). However, the baseline values in the present study for knee joint distraction were higher (better condition) than in the previous uncontrolled study (54 ± 20 and 45 ± 16 points, respectively).

This difference at baseline and similarity for 1 year’s outcome was also seen for VAS pain. This may be explained by the fact that in the present randomized controlled trial, patients were in general practice considered for HTO, whereas in the previous prospective uncontrolled knee joint distraction study, patients were treated with a new experimental technique and only severe end-stage knee OA was considered. This difference in disease activity was reflected by a difference in disease severity (joint damage). In the knee joint distraction population of the uncontrolled study, 55 % had Kellgren & Lawrence (KLG) grade 3 at baseline and 10 % had grade 4. In the present study, 50 % had grade 3 and 5 % had grade 4. The presently treated group of patients treated with knee joint distraction had medial compartment osteoarthritis and participated on a higher level in daily society than the patients in the previously uncontrolled study. These results show that even in younger patients, who undertake high-demanding activities for the knee (e.g. recreational sports), knee joint distraction treatment may lead to clinical relevant improvement.

A number of previous prospective studies utilizing valgus-producing opening-wedge HTO using KOOS and VAS pain scores have been carried out [8, 14, 32, 40, 43]. In these studies, the KOOS score was between 60 and 63 points [8, 32, 40], and VAS pain was between 21 and 25 mm at 12 months [14, 43]. Clinical outcome of the HTO treated patients in the present study was 68 ± 19 points for KOOS and 27 ± 23 mm for VAS pain, which is comparable to those of previous studies. Also the rate of superficial (2.2 %) and deep (2.2 %) wound infections in the HTO group is in line with the literature, which showed a rate of 1–9 % for superficial wound infections and 0.5–4.7 % for deep infections [3]. As expected, the rate of pin tract infections in the knee joint distraction group was relatively high. In general, external fixation infection varies from as low as 3 % to over 80 %, depending of the used external fixator and the various definitions of pin site infections [16]. All patients observed with a pin tract infection were adequately treated with antibiotics, but minimizing such infections is desirable.

In general, it is difficult to judge differences in burden between both treatments. Not unexpectedly, external fixation causes patient discomfort, and the knee joint distraction group were asked about this. In general, activities of daily living, such as showering, walking and sleeping, did not give much discomfort. Patients having a clerical job were even able to continue the work during the distraction treatment. Most patients indicated that they would undergo knee joint distraction treatment again. Moreover, some patients subsequently received knee joint distraction of their contralateral osteoarthritic knee some time later.

**Fig. 6:**
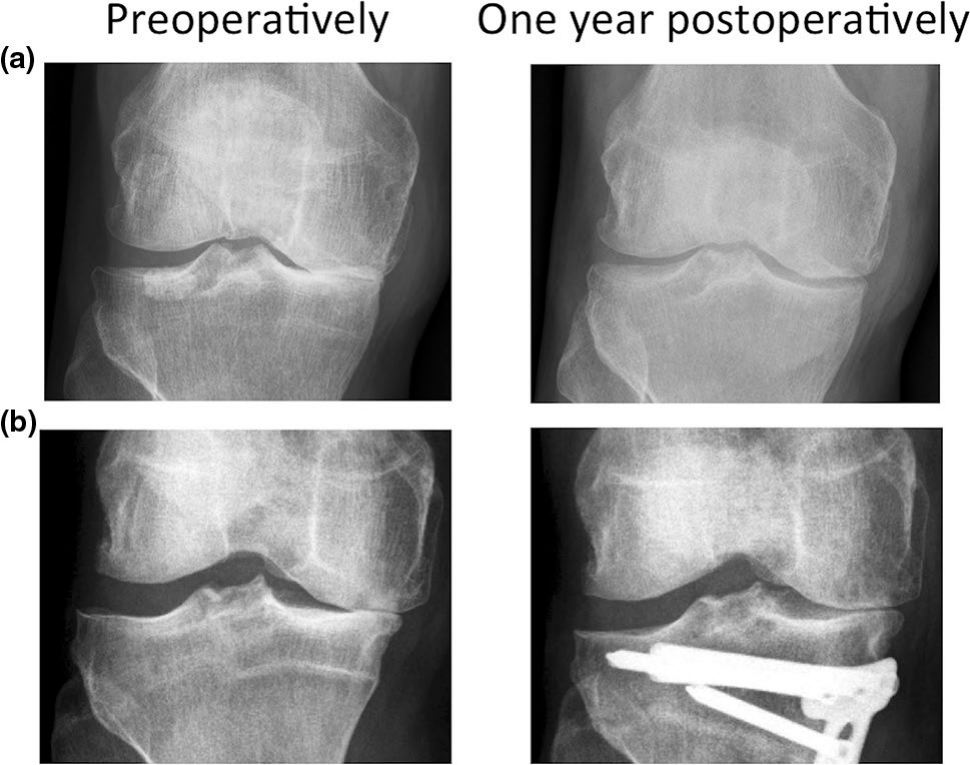
**a** Radiograph preoperatively (left) and 1 year post-operatively (right) of a representative patient treated with knee joint distraction. **b** Radiograph preoperatively (left) and 1 year post-operatively (right) of a representative patient treated with HTO

For both HTO and knee joint distraction, a significant increase in radiographic JSW was observed (see Fig. 6 for representative examples). For knee joint distraction, this was not confined to the medial (affected) compartment, but also, although less pronounced, in the lateral compartment. In case of knee joint distraction, the JSW on weight-bearing radiographs is considered to represent thickness of resilient cartilage tissue (weight-bearing radiographs), since it is not anticipated that knee joint distraction alters the alignment of the knee joint significantly. This cartilage regenerating capacity was supported by MRI data [24, 25]. In case of HTO, the change from varus alignment to valgus alignment may have created a joint space at the medial site not representing cartilage thickness per se. Moreover, this shift in alignment resulted in a decrease in JSW at the lateral side. Earlier studies showed similar widening of the medial compartment (ranging from +0.4 mm till +1.1 mm) and a decrease in the lateral compartment [5, 25, 37]. Longer-term follow-up and MRI analyses (first follow-up after 2 years because of plate interference) in the present study will reveal what the outcome on cartilage regeneration between the two approaches will be.

This trial had incomplete blinding and lack of a placebo. However, numerous surgical practices have evolved into standard of care without being randomized against placebo/sham intervention [22]. Adding a placebo-control arm to our RCT would hardly be ethical: active, relatively young patients with symptomatic medial compartmental OA would be deprived of standard operative care for several years. Secondly, the homeostatic joint mechanisms implicated in the effects of joint distraction are clearly understood and described in vitro human cartilage models and in vivo canine models of knee joint distraction [4, 46]. Thirdly, both treatments have shown persistent clinical benefit up till 5 years after treatment [9, 23]. Finally, an eventual placebo effect would be expected to be equal in both groups.

For medial compartment OA, a load-bypassing knee support system has been reported (KineSpring System). Although initial studies reported joint pain and function improvement [29], later studies reported serious risks including development of intra- and extra-articular metallosis, medial joint capsule and medial collateral ligament/medial joint instability after device removal [39]. Furthermore, studies comparing the KineSpring with HTO are lacking. Other treatment options would be unicompartimental knee arthroplasty or TKA, the latter being less favourable in our relative young patients. Register studies [21, 35] showed a higher rate of aseptic loosening of unicompartimental knee arthroplasties, so it is not advised to perform this procedure in patients younger than 55 years [28]. International register studies described even more unfavourable results in patients aged below 65 years after TKA [36]. Clearly, a TKA at relatively young age should be postponed as long as possible to prevent patients from revision surgery.

This study has some limitations, as there is a high heterogeneity in the patient’s parameters at baseline (Table 1). In the HTO group, 51 % of the patients had OA of grade 3 and even 9 % of the patients had OA of grade 4. In the literature, the ideal candidate for HTO has a maximum KLG of 2, and it is described that a KLG (3 or 4) is associated with a poorer clinical outcome and as a risk factor for conversion to arthroplasty 10 years after HTO [41, 42, 44]. On the other hand, in a study of 91 patients (average age patients 50.4 years) with KLG 3 and 4, the 5-year knee survival rate was 95.2 % [38]. Other factors negatively influencing the outcome of HTO are female gender and obesity [41, 44]. Noteworthy is the difference in female gender between the HTO group and the knee joint distraction group (40 vs. 27 %). One could imagine that this relative difference influenced the outcome in the HTO group. Furthermore, in the HTO group, two patients were included who had a previous HTO that led to an under-correction (persistent varus malalignment). For the knee joint distraction group, there was the same heterogeneity; however, since this is a relatively new treatment, specific patient parameters influencing clinical outcome currently remain unknown. Nevertheless, in our study, all patients had a clear varus deformity in the proximal tibia with a varus malalignment and medial compartment OA resistant to conservative treatment with no other joint-preserving treatment options and would have been considered in general practice for HTO.

In general, it may be concluded that knee joint distraction may be considered in case of varus malalignment as an alternative to HTO. After the short follow-up time in this study, choosing between HTO and knee joint distraction will be based on personal preference, based on experience, and personal ‘belief’. Burden of patients should be considered as well, leaving space for improvement of the distraction device. Midterm and long-term results of knee joint distraction treatment are mandatory to make an evidence-based decision.

## Conclusion

Knee joint distraction is an effective and valuable treatment option in patients with knee OA, even with a varus deviation of up to 10°, providing structural and clinical improvement in this relative young patient category.
